# Supplementary material for: Flow Ripple Reduction in Reciprocating Pumps by Multi-Phase Rectification
Source: Sensors (Basel). 2023 Aug 5;23(15):6967. doi: 10.3390/s23156967 (PMC10422673; doi:10.3390/s23156967)
Supplement: Supplementary file 1 [file sensors-23-06967-s001.zip › sensors-2292164-supplementary.pdf]

sensors

MDPI

Supplementary information

Flow Ripple Reduction in Reciprocating Pumps by Multi-Phase Rectification

Gürhan Özkayar [1,*], Zhilin Wang [1], Joost Lötters [1,2], Marcel Tichem [1] and Murali Krishna Ghatkesar [1,*]

[1] Department of Precision and Microsystems Engineering, Delft University of Technology, 2628 CD, Delft, The Netherlands; z.wang-20@tudelft.nl (Z.W.)
[2] Bronkhorst High-Tech B.V., 7261 AK Ruurlo, The Netherlands
* Correspondence: g.ozkayar@tudelft.nl (G.Ö.); m.k.ghatkesar@tudelft.nl (M.K.G.)

Supplementary information

The following matlab code can be used to estimate the ripple factor for n-phase rectifier.

```
%The MATLAB code used for calculating theoretical values.
n = input("Enter pump amount:");
f = 1; %frequency [Hz]
a = input("Enter flow rate(ul/min):"); %flow rate [ul/min]
t = 0:1:720; %time
phi = zeros(); %first value for phase
sum1 = zeros(); %first value for sum
subplot(2,1,1);
for i = 1:n
phi(i,:)= (i − 1)*360/n; %phase shift in degrees (360° = 2*pi)
sum1 = a*sind(f*t-phi); %calculation of sum with varying phases
sum1(sum1 < 0) = 0; %HWR part, negative values are converted into zero
plot(t,sum1(i,:),'linewidth',2) %plotting each individual pump
hold on
legend("Pump with " + phi + "° phase shift",'FontSize',12); %legends of each phase shifted pump
lgd = legend;
lgd.NumColumns = 5;
out = sum(sum1,1); %sum1 has n rows, they are converted into one row array as a summation
out_avg = mean(out);
out_rms = rms(out);
out_max = max(out);
ff_out = out_rms./out_avg;
rf_out = real(sqrt((ff_out).^2 − 1));
end
plot(t,out,'k--','DisplayName','Sum','linewidth',3)
hold off
title("Multi-phase rectification with phase shifted parallel pumps, " + n + " pump(s)",'FontSize',24);
xlabel('Phase (°)','FontSize',20);
ylabel('Flow rate (\mul/min)','FontSize',20);
```

```
set(gca,’FontSize’,18)
axis([xlim 0 720]); %dependent on t value
axis([ylim −1 (a + (a*n)/3)]); %maximum y value is dependent on a and n values
grid on
grid minor
hold on
subplot(2,1,2);
Qavg(n) = out_avg
Qrms(n) = out_rms
Qmax(n) = out_max
RF(n) = rf_out
title(‘Q_A_V_G, Q_R_M_S, and RF_f_l comparison for different rectification levels’,’FontSize’,24)
xlabel(‘Rectification level’,’FontSize’,20)
yyaxis left
plot(n,Qavg(n),’k-o’,n,Qrms(n),’k-square’,n,Qmax(n),’k-
^’,’MarkerFaceColor’,’k’,’MarkerSize’,8,’linewidth’,1.5)
ylabel(‘Flow Rate (\mul/min)’,’FontSize’,20)
set(gca,’FontSize’,18)
axis([xlim 0.5 n + 1]);
axis([ylim −1 (a + (a*n)/3)]);
hold on
yyaxis right
plot(n,RF(n),’r-v’,’MarkerFaceColor’,’r’,’MarkerSize’,8,’linewidth’,1.5)
ylabel (‘Fluidic Ripple Factor’,’FontSize’,20)
axis([xlim 0.5 n + 1]);
axis([ylim 0 1.5]);
grid on
grid minor
left_color = [0 0 0];
right_color = [1 0 0];
%set(figure,’defaultAxesColorOrder’,[left_color; right_color]);
hold on
legend({‘Q_a_v_g’, ‘Q_r_m_s’, ‘Q_m_a_x’,
‘RF_f_l’},’Orientation’,’horizontal’,’Location’,’northwest’,’FontSize’,18)
clear phi
%A MATLAB app is also created for quick calculations of flow ripples. The application is available
here:
https://github.com/gurozkayar/MultiPhase_Rectification_Tool_For_Reciprocating_Micropumps/tre
e/v1.0.0 (accessed on 2 August 2023).
```
